# Hypoxic glioma‐derived extracellular vesicles harboring MicroRNA‐10b‐5p enhance M2 polarization of macrophages to promote the development of glioma

**DOI:** 10.1111/cns.13905

**Published:** 2022-09-02

**Authors:** Bingzhen Li, Cheng Yang, Zhanpeng Zhu, Hao Chen, Bin Qi

**Affiliations:** ^1^ Department of Neurosurgery the First Hospital of Jilin University Changchun P. R. China

**Keywords:** extracellular vesicles, glioma, microRNA‐10b‐5, neuronal precursor cell expressed developmentally downregulated 4‐like, phosphatidylinositol‐4, 5‐bisphosphate 3‐kinase

## Abstract

**Introduction:**

The delivery of biomolecules by tumor cell‐secreted extracellular vesicles (EVs) is linked to the development of glioma. Here, the present study was implemented to explore the functional significance of hypoxic glioma cell‐derived EVs carrying microRNA‐10b‐5 (miR‐10b‐5p) on glioma with the involvement of polarization of M2 macrophages.

**Methods:**

EVs were isolated from hypoxia‐stimulated glioma cells, and their role in polarization of M2 macrophages was studied by co‐culturing with macrophages. miR‐10b‐5p expression in glioma tissues, glioma‐derived EVs, and macrophages co‐cultured with EVs was characterized. Interaction among miR‐10b‐5p, NEDD4L, and PIK3CA was analyzed. The macrophages or glioma cells were transfected with overexpressing plasmid or shRNA to study the effects of miR‐10b‐5p/NEDD4L/PIK3CA on M2 macrophage polarization, and glioma cell proliferation, migration, and invasion in vitro and in vivo*.*

**Results:**

Promotive role of hypoxia‐stimulated glioma‐derived EVs in macrophage M2 polarization was confirmed. Elevation of miR‐10b‐5p occurred in glioma tissues, glioma‐derived EVs and macrophages co‐cultured with EVs, and stimulated M2 polarization of macrophages. NEDD4L was a target gene of miR‐10b‐5p. Overexpression of NEDD4L could inhibit PI3K/AKT pathway through increase in ubiquitination and degradation of PIK3CA. Hypoxic glioma‐derived EVs harboring upregulated miR‐10b‐5p triggered an M2 phenotype in macrophages as well as enhanced aggressive tumor biology of glioma cells via inhibition of PIK3CA/PI3K/AKT pathway by targeting NEDD4L.

**Conclusions:**

In summary, miR‐10b‐5p delivered by hypoxic glioma‐derived EVs accelerated macrophages M2 polarization to promote the progression of glioma via NEDD4L/PIK3CA/PI3K/AKT axis.

## Graphical abstract

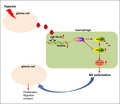

## 1 INTRODUCTION

As the most prevalent primary malignant brain tumors,^1^, ^2^ glioma is characterized by heterogeneous angiogenesis and invasiveness.^3^ Gliomas arise from glial cells of the central nervous system which can be divided into two histopathological subgroups: low‐grade glioma and high‐grade glioma.^4^ Glioblastoma multiforme and anaplastic glioma are recognized as the most common gliomas, which account for more than 50% and more than 10% of the total gliomas, respectively.^5^ There are many therapeutic options for glioma including surgery, radiation, and, temozolomide, but this brain tumor is susceptible to temozolomide resistance, immunosuppression, or even recurrence.^6^, ^7^, ^8^, ^9^ Non‐tumor cells are a component of glioma, and most of them are tumor‐related macrophages, which originate from peripheral or on behalf of microglia cells in the brain, supporting the progression of tumor cells.^10^ Environmental stimuli and metabolites produced within the cell affect macrophage activation, and activated macrophages can be polarized into M1 and M2 macrophages.^11^ M2 macrophages are the most important immune cells in the glioma microenvironment, whose activation contributes to the development of glioma.^12^ Thus, targeting key molecules that modulate the polarization of M2 macrophages is a necessary for improving treatment of glioma.^13^

Extracellular vesicles (EVs) are membrane structures derived from heterogeneous cells composed of exosomes and microvesicles, which are regarded as a mechanism for cell‐to‐cell communication.^14^ EVs have huge impacts on tumor development of several cancers including glioma,^15^ which function as the most promising biomarkers for glioma treatment.^16^ More importantly, EVs carrying microRNA (miRNAs) from donors to recipient cells can trigger profound effect in tumor microenvironment.^17^ miRNAs are involved in various biological processes, such as cell proliferation, migration, invasion, and apoptosis and serve as an appealing therapeutic targets for cancer treatment.^18^, ^19^ For example, elevation of microRNA‐10b‐5p (miR‐10b‐5p) has been confirmed in glioma tissues and hypoxia‐stimulated glioma‐derived EVs.^20^, ^21^ Moreover, neuronal precursor cell expressed developmentally downregulated 4‐like (NEDD4L) was a target gene of miR‐10b‐5p based on the starBase prediction website in present study. NEDD4L belongs to Nedd4 family, which is a ubiquitin‐protein ligase with a unique modular structure and targets specific proteins for ubiquitination.^22^ The inhibition of NEDD4L has been reported to be related to the aggressive progression and dismal prognosis of malignant glioma.^23^ Phosphatidylinositol‐4, 5‐Bisphosphate 3‐Kinase (PIK3CA) plays a crucial role in PI3K‐related cancer progression, which can be catalyzed through polyubiquitination by NEDD4L, resulting in its proteasome‐dependent degradation.^24^ Furthermore, the mutations of PIK3CA triggers excessively activated PI3K/AKT pathway, which may be one of possible mechanisms of pathogenesis of glioma.^25^

Therefore, we hypothesized that hypoxia‐stimulated glioma‐derived EVs harboring miR‐10b‐5p may affect the M2 polarization of macrophages through NEDD4L/PIK3CA/PI3K/AKT axis. Therefore, this study was conducted to verify this hypothesis, which may provide new possible targets for anti‐glioma therapy.

## 2 METHODS

### 2.1 Ethical approval

The study was conducted under the approval of the Ethics Committee of The First Hospital of Jilin University and conformed to the *Declaration of Helsinki*. All participants or their guardians signed informed consent. Animal experiments were implemented in compliance with recommendations in the Guide for the Care and Use of Laboratory Animals of the *National Institutes of Health*.

### 2.2 Bioinformatics analysis

Glioma miRNA expression dataset GSE65626 deposited in GEO database was harvested, including three normal samples and three tumor samples. With the normal samples as controls, the R language “limma” package was adopted for differential analysis, and the false discovery rate method was utilized to correct the differential *p*‐value. |logFC| > 2 and adj.*p*.val < 0.05 were employed as screening criteria for obtaining differential miRNAs. miRNAs expressed in blood EVs were searched through EVmiRNA database, and miRNAs with expression value greater than 2000 were chosen as candidate miRNAs. The starBase database was applied to predict targeted binding sites of miR‐10b‐5p and NEDD4L. PIK3CA‐related genes were retrieved in the GeneMANIA database to acquire the network map of related genes. The “clusterprofiler” package was utilized to perform KEGG pathway enrichment analysis on PIK3CA‐related genes.

### 2.3 Study subjects

In this study, 40 glioma specimens were attained from patients who were diagnosed as primary glioma for the first time without basic diseases, including infection, inflammation, hepatitis, and diabetes in The First Hospital of Jilin University from 2016 to 2018. Fifteen non‐cancerous brain tissues from patients with trauma, epilepsy, and vascular malformations were collected after surgery. Surgery was performed prior to chemotherapy or radiotherapy. The glioma specimens consisted of 17 cases of the World Health Organization (WHO) grade II diffuse astrocytoma, 14 cases of WHO grade III anaplastic astrocytoma, and 9 cases of WHO grade IV glioma blastoma.

### 2.4 Immunohistochemistry

The brain tissues of 40 glioma specimens and 15 non‐glioma brain tissues were sectioned. Paraffin tissue sections were dewaxed and dehydrated in gradient alcohol. After sections were treated with 3% H_2_O_2_ for 20 min, antigen retrieval was carried out. Then, sections were incubated with normal goat serum (C‐0005, Haoran Biotechnology Co., Ltd) at ambient temperature for 20 min and probed with primary rabbit anti‐human NEDD4L (ab46521, 1: 100), PIK3CA (ab40776, 1: 500), and Ki67 (ab16667, 1: 200) overnight (4°C). Sections were reprobed with goat anti‐rabbit immunoglobulin G (IgG) as the secondary antibody at 37°C for 20 min, followed by incubation with streptavidin labeled by horseradish peroxidase (0343‐10000U; Imunbio Biotechnology Co., Ltd.) for 20 min at 37°C. All antibodies were obtained from Abcam. Sections were subjected to peroxidase substrate diaminobenzidine (DAB; ST033; Weijia). All sections were counterstained with hematoxylin for 1 min, returned blue with 1% ammonia, dehydrated in ascending series of alcohol, cleared with xylene, and sealed with neutral resin. Sections of randomly selected 5 high‐power field of view were observed by experienced pathologists and photographed under a microscope.

### 2.5 Cell culture and transfection

Human GBM cell lines U87 and A172 and macrophage U937 cell line were provided by the Cell Bank of the Chinese Academy of Sciences (Beijing, China). U87 and A172 cells were cultured in a Dulbecco's modified Eagles Medium (DMEM) containing 10% fetal bovine serum (FBS), 10 μg/ml streptomycin, and 100 U/ml penicillin (Gibco). U937 cells were incubated in Roswell Park Memorial Institute (RPMI)‐1640 medium (Thermo Fisher Scientific) supplemented with 10% FBS. U937 cells were cultured with 100 ng/ml phorbol 12‐myristate 13‐acetate (PMA; Sigma‐Aldrich) for 24 h in vitro to induce differentiation of U937 cells into macrophages. Cells were co‐cultured with 1 μg/ml EV fluid.

In order to simulate a hypoxic environment, U87 and A172 cells were cultured in a fresh EV‐free DMEM and then incubated in a hypoxic oxygen incubator (Forma Scientific Inc.) for 18–24 h, which containing 5% CO_2_, 10% H_2_, 85% N_2,_ and <0.5% O_2_. The untreated cells were cultured in the same way in a 37°C normoxic incubator containing 5% CO_2_.

LV5‐GFP (overexpressing of gene vector) and pSIH1‐H1‐copGFP (silencing of gene vector) were utilized for lentivirus packaging. Lentivirus vectors harboring mimic‐negative control (NC), miR‐10b‐5p mimic, inhibitor‐NC, miR‐10b‐5p inhibitor, overexpressed (oe)‐NEDD4L, oe‐NC, shRNA (sh)‐NC, sh‐NEDD4L‐1, sh‐NEDD4L‐2, or oe‐PIK3CA were infected into 293 T cells for 48 h, followed by collection of supernatant, mimic‐NC, or miR‐10b‐5p mimic plasmid were transduced into U87 cells. Lentivirus vectors harboring mimic‐NC, miR‐10b‐5p mimic, inhibitor‐NC, miR‐10b‐5p inhibitor, vector + mimic‐NC, oe‐NEDD4L + mimic‐NC, oe‐NEDD4L + miR‐10b‐5p mimic, oe‐NEDD4L, oe‐NC, sh‐NC, sh‐NEDD4L‐1, sh‐NEDD4L‐2, oe‐PIK3CA, or oe‐NEDD4L + oe‐PIK3CA plasmids were infected into U937 cells. Cells were trypsinized, prepared into cell suspension (5 × 10^4^ cells/ml), seeded into 6‐well plates (2 ml/well), and cultured at 37°C overnight for 48 h. The transfection sequences and plasmids were purchased from Shanghai GenePharma. All plasmids were used at a concentration of 50 ng/ml.

### 2.6 Extraction and identification of EVs derived from glioma cells

Glioma cells were cultured overnight in EV‐free DMEM. Upon 80%–90% cell confluence, the collected supernatant was centrifuged at 2000 *g* (20 min) at 4°C, and the obtained supernatant was subjected to high‐speed centrifugation at 10,000 *g* (1 h; 4°C). Pellet was resuspended in serum‐free DMEM containing 25 mM HEPES (pH = 7.4) and subjected to high‐speed centrifugation. Following supernatant removal, the precipitated was stored at −80°C.

EVs were characterized by a transmission electron microscopy (TEM). Dynamic light scattering was applied to detect the diameter of EVs using the Zetasizer Nano‐ZS90 instrument (Zetasizer Nano‐ZS90; Malvern) with an excitation light wavelength λ = 532 nm. The EV samples were diluted with 0.15 M NaCl to the appropriate optical signal detection level (1, 50).

The EV particles were dissolved in radio‐immunoprecipitation assay (RIPA) lysis buffer, and the protein was quantified using a bicinchoninic acid (BCA) kit (Thermo Fisher Scientific). EVs were analyzed using immunoblotting with the following antibodies (Abcam) to CD9 (ab92726, 1: 2000), CD63 (ab216130, 1: 2000), TSG101 (ab125011, 1: 1000), and Calnexin (ab22595, 1: 100).

### 2.7 Uptake of EVs

The cell slide was put above the culture dish and U937 cells were placed in the culture dish for cultivation. When cell density reached 50%, the slide was removed, washed slowly with PBS 3 times, soaked for 30 min with 4% paraformaldehyde at ambient temperature, and permeated with 2% Triton X‐100 for 15 min, followed by blocking with 2% bovine serum albumin (BSA) for 45 min. After discarding the blocking solution, PHK67‐labeled glioma cell‐derived EVs were added to cells for 24 h of co‐culture. Then, the slide was stained with 4′6‐diamidino‐2‐phenylindole (DAPI; 2 μg/ml), and mounted with the fluorescence measured under an upright fluorescence microscope.

### 2.8 Flow cytometry

To detect CD11b^+^CD163^+^ macrophages, cells were stained with anti‐CD163‐PE (BD Biosciences) and anti‐CD11b‐APC (eBioscience). Flow cytometry was performed using BD Accuri C6 flow cytometry (BD Biosciences).

### 2.9 RT‐qPCR

Total RNA from tissues was extracted using TRIzol reagent (16096020, Thermo Fisher Scientific). For the detection of mRNA, reverse transcription was performed using reverse transcription kit (RR047A, Takara) to generate complementary DNA (cDNA), while for miRNA, the PolyA tailing detection kit (B532451, Sangon Biotech company) (containing universal PCR primer R and U6 universal PCR primer R) was applied. SYBR® Premix Ex TaqTM II (Perfect Real Time) kit (DRR081, Takara) was applied to add samples. RT‐qPCR assay was developed using fluorescence‐based quantitative PCR instrument (ABI 7500, Applied Biosystems). The primers were designed by Sangon (Table S1). The relative expression of target genes was measured by 2^−ΔΔCt^ method normalized to U6 and glyceraldehyde‐3‐phosphate dehydrogenase (GAPDH).

### 2.10 Immunoblotting

Total protein was extracted, electrophoresed, and then electroblotted to polyvinylidene fluoride membranes (IPVH85R; Millipore). Immunoblotting was implemented with diluted primary antibodies, rabbit anti‐human antibodies to NEDD4L (ab46521, 1: 1000; Abcam), PIK3CA (ab40776, 1: 2000; Abcam), iNOS (ab213987, 1: 1000; Abcam), Arg‐1 (MAB5868‐SP; ab41525, 1: 25; R&D systems), phosphorylated AKT (ab81283, 1: 500; Abcam), AKT (ab8805, 1: 500; Abcam), and GAPDH (ab706699, 1: 3000; Abcam) as well as secondary antibody, goat anti‐rabbit antibody to immunoglobulin G (IgG; ab205718, 1: 5000; Abcam) labeled by HRP. Developing solution was applied for visualization. Protein gray scale analysis was performed using ImageJ 1.48u software (National Institutes of Health).

### 2.11 Enzyme‐linked immunosorbent assay (ELISA)

The serum from nude mice or cell supernatant of each group was collected. The expression of the TNF‐α and IL‐10 was measured by ELISA kit (MTA00B, DY417‐05, R&D systems). The absorbance (A) value of each well at 450 nm was measured using a versatile microplate reader (Synergy 2, BioTek, Biotek Winooski), and the standard concentration was taken as the *x*‐axis and the A value as *y*‐axis. The regression equation of the standard curve was calculated. The sample A value was substituted into the equation to calculate the target protein concentration in the sample.

### 2.12 Dual‐luciferase reporter gene assay

The wild type (WT) and mutant type (MUT) sequence of NEDD4L 3′‐untranslated region (3′UTR) were cloned into PmirGLO reporter (PmirGLO‐NEDD4L‐Wand and PmirGLO‐NEDD4L‐MUT). PmirGLO‐NEDD4L‐Wand or PmirGLO‐NEDD4L‐MUT (5’‐AGUAUUACGACUCUACAAAUGU‐3′) were co‐transfected with miR‐10b‐5p mimic or NC‐mimic into HEK293T cells for 48 h. Luciferase activity was measured using Dual‐Luciferase® Reporter Assay System (E1910; Promega). Luciferase activity was directly measured by the ratio of firefly luciferase activity to renilla luciferase activity.

### 2.13 5‐ethynyl‐2′‐deoxyuridine (EdU) assay

Cells were seeded in 24‐well plates with 3 duplicate wells for each group. The EdU assay was performed with an EdU assay kit. Cells were sealed and observed under a fluorescence microscope (FM‐600, Shanghai Pudan Optical Instrument Co., Ltd.). Six to ten fields were randomly selected, and the number of positive cells in each visual field was recorded.

### 2.14 Transwell assay

A Transwell chamber (8 mm aperture; Corning) pre‐coated with Matrigel or without was used for cell migration and invasion detection in 24‐well plates.^26^ Invasive and migrated cells were observed under an inverted fluorescence microscope (TE2000, Nikon) with five fields of view randomly selected for taking the mean value.

### 2.15 Ubiquitination assay

Plasmids with His‐tagged ubiquitin, Flag‐NEDD4L, and HA‐PIK3CA were transfected into HEK293T cells. In order to enrich the ubiquitinated protein, the cell lysate was incubated with Talon beads (GE Healthcare). After incubating for 2 h at 4°C, ubiquitinated proteins were eluted with 20 ml of eluent (5 × SDS buffer, 0.05 M ethylenediaminetetraacetate [EDTA], and 1% SDS lysis buffer). Ubiquitinated proteins were subjected to immunoblotting with PIK3CA (ab40776; 1: 1000; Abcam) or HA antibody (ab9110; 1: 2000, Abcam).

### 2.16 Co‐immunoprecipitation (Co‐IP)

Protein samples were prepared by adding HEPES lysis buffer (20 mM MHEPES, 200 mM NaCl, 1 mM EDTA, 1 mM EDTA, 1% Triton x‐100, 5 mM pyrophosphate, 20 mM chap‐glycerophosphate, and 50 mM sodium fluoride) supplemented with protease inhibitor (Roche). Cell lysis buffer was centrifuged with supernatant collected and incubated with antibodies. Then, the sample was treated with protein A/G agarose beads (434341; Thermo Fisher Scientific) for 2 h. Proteins in agarose beads were eluted with 2× SDS sample buffer and assessed by immunoblotting. Primary antibody used were as follows: IP: Flag (ab205606, 1: 30, Abcam) and His (ab18184, 1: 200, Abcam); immunoblotting: PIK3CA (ab40776, 1: 1000, Abcam), HA antibody (ab9110, 1:2000, Abcam), GAPDH (ab706699, 1: 3000, Abcam), Flag (ab205606, 1: 1000, Abcam), and Ub (ab134953, 1: 1000, Abcam).

### 2.17 Tumor xenografts in nude mice

Twenty‐four male nude mice aged 5 weeks were selected for our experiment. Macrophages were treated with PBS, and EVs from glioma cells treated with hypoxia and transfected with mimic‐NC (H‐EV‐mimic‐NC) or miR‐10b‐5p mimic (H‐EV‐miR‐10b‐5p mimic) for 48 h. Glioma cells U87 (1 million cells/mouse) were mixed with macrophages of different conditions (200,000 cells/mouse) and injected subcutaneously into nude mice. Then, PBS or H‐EV‐mimic‐NC or H‐EV‐mimic‐NC was injected into nude mice through tail vein every 3 days. A vernier caliper was utilized to record the short diameter (a) and long diameter (b) of the tumor every 5 days after implantation. Tumor volume was calculated based on *π* (*a* ^2^ *b*)/6. After 25 days, nude mice were euthanized after anesthetization with pentobarbital sodium (100 mg/kg; P3761; Sigma). The tumors were dissected and isolated. Tumor weight was measured with a balance.

### 2.18 Statistical analysis

The data representative of three independent experiments in triplicate were processed using SPSS 21.0 statistical software (IBM Corp.) and were expressed summarized by mean ± standard deviation. Data distribution was evaluated by Shapiro–Wilk test, and all data were normally distributed. Paired data in compliance with normal distribution and homogeneity of variance between two groups were compared using unpaired *t*‐test. Comparisons among multiple groups were conducted by one‐way analysis of variance with Tukey's post hoc test. A value of *p* < 0.05 indicated significant difference.

## 3 RESULTS

### 3.1 Hypoxic glioma cell‐derived EVs induce M2 macrophage polarization

**FIGURE 1:**
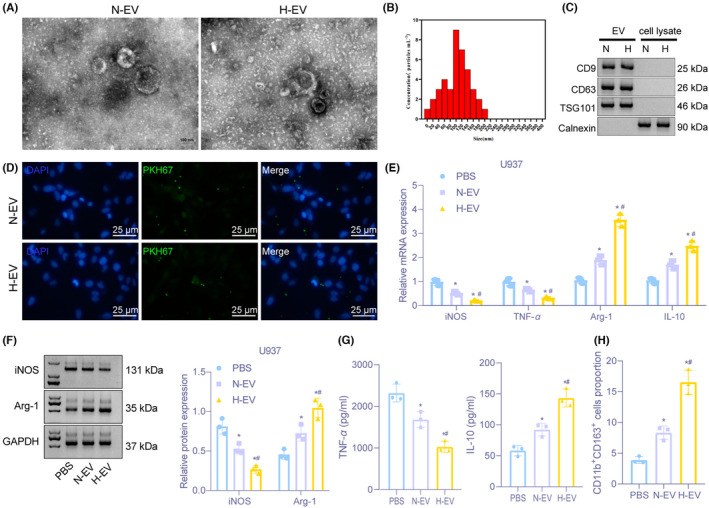
Hypoxic glioma cell‐derived EVs promote M2 macrophage polarization. (A) Images of EVs observed under a TEM (Scale bar = 100 nm). (B) Diameter of EVs detected by dynamic light scattering. (C) The expression of CD9, CD63, TSG101, and calnexin on the surface of EVs determined by Immunoblotting. (D) Detection of the uptake of EVs from normoxic and hypoxic glioma cells by macrophages U937 through immunofluorescence (400×). (E) The mRNA expression of iNOS, TNF‐α, Arg‐1, and IL‐10 assessed by RT‐qPCR after EV treatment. (F) Immunoblotting of protein expression of iNOS and Arg‐1 in macrophages U937 after EV treatment. (G) The expression of TNF‐α and IL‐10 in supernatant of macrophages U937 evaluated by ELISA assay. (H) The proportion of CD11b^+^CD163^+^ cells in U937 cells examined by flow cytometry. *p < 0.05 versus macrophages treated with PBS. ^#^ *p* < 0.05 versus macrophages treated with normal oxygen‐induced glioma cell‐derived EVs. The experiment was repeated three times

To study the impact of hypoxia on macrophages in the glioma microenvironment, ultracentrifugation was used to separate the EVs in the supernatant of U87 cells under normoxic and hypoxic conditions. TEM observation (Figure 1A) displayed that EVs under normoxic and hypoxic conditions were round or oval membranous vesicles with similar morphology, and their sizes were revealed to be at 30–200 nm, as reflected by dynamic light scattering (Figure 1B). Immunoblotting presented significantly increased expression in EV surface markers CD9, CD63, and TSG101, without calnexin expression (Figure 1C), confirming successful extraction of EVs. After co‐cultivation of PKH67‐labeled normoxic and hypoxic EVs with macrophage U937 cells, the uptake of PKH67 by macrophages was very obvious, indicating the successful uptake of EVs by macrophage U937 cells (Figure 1D).

To investigate the function of glioma cell‐derived EVs on macrophage polarization, normal oxygen‐treated glioma cell‐derived EVs (N‐EV) or hypoxia‐treated glioma cell‐derived EVs (H‐EV) were co‐cultured with macrophages for 24 h. RT‐qPCR showed relative to PBS and N‐EV, M2 macrophage markers’ (Arg‐1 and IL‐10) levels were significantly increased, but M1 macrophage markers’ (iNOS and TNF‐α) levels were reduced in macrophages treated with H‐EV (Figure 1E), and consistent trends were obtained by immunoblotting (Figure 1F) and ELISA (Figure 1G). Flow cytometry illustrated that the ratio of CD11b^+^CD163^+^ cells in macrophage treated with H‐EV was elevated significantly in comparison with PBS or N‐EV treatment (Figure 1H). The above results demonstrated that hypoxia‐induced glioma cell‐derived EVs could promote M2 macrophage polarization.

### 3.2 Hypoxic glioma cell‐derived EVs carrying miR‐10b‐5p promote M2 macrophage polarization to induce glioma development

**FIGURE 2:**
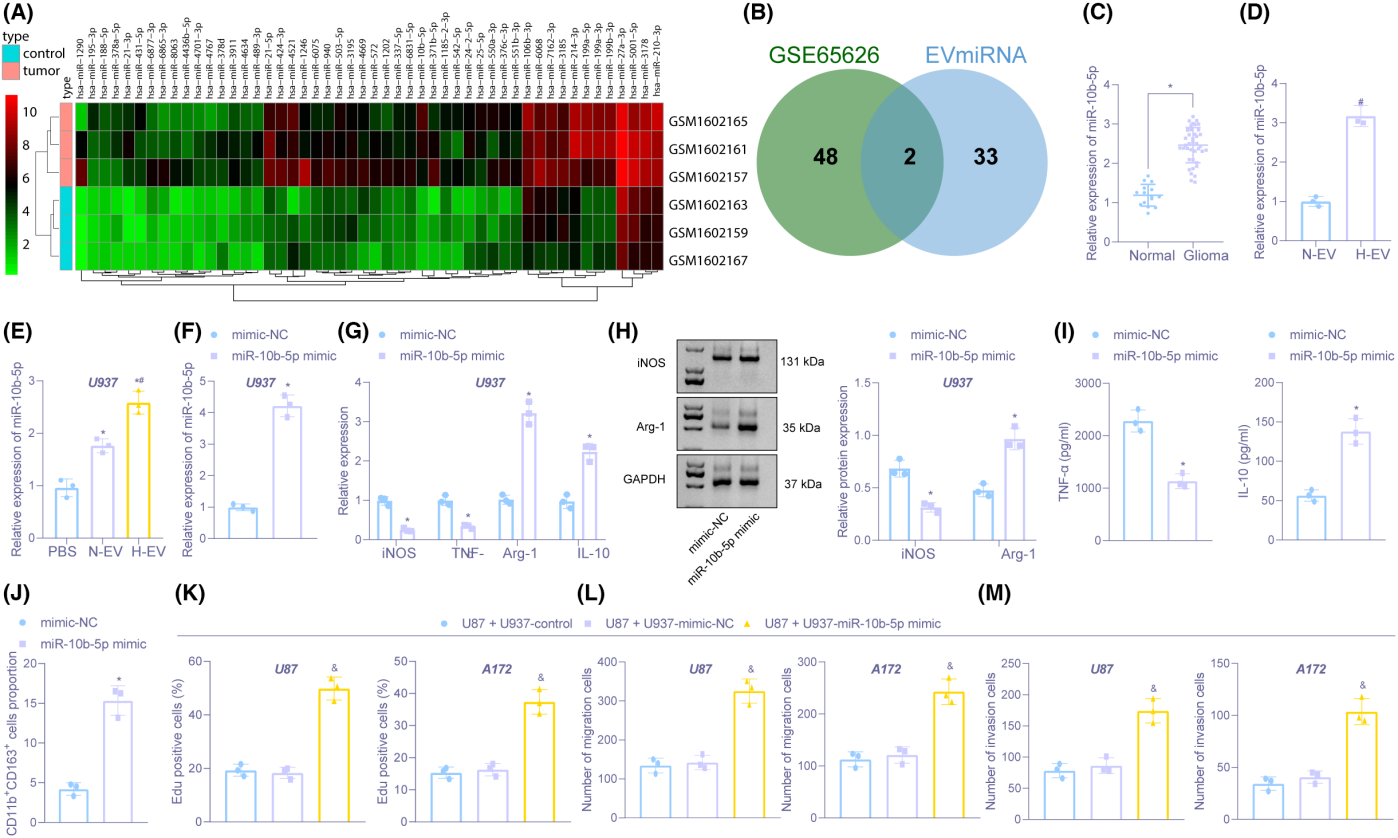
Hypoxic glioma cell‐derived EVs harboring miR‐10b‐5p facilitate M2 polarization of macrophages, thus increasing glioma development. (A) Heatmap of differential miRNAs in glioma‐related miRNA expression microarray. The x‐axis represents the sample number, the y‐axis represents the miRNA name, and the upper right histogram is color scale. (B) Intersection of differential miRNAs with miRNAs in blood EVs in EVmiRNA database. The middle part is the intersection of two sets of data. (C) The expression of miR‐10b‐5p in brain tissues of 40 cases of glioma and 15 cases of non‐glioma examined by RT‐qPCR. (D) The expression of miR‐10b‐5p in N‐EV and H‐EV determined by RT‐qPCR. (E) The expression of miR‐10b‐5p in U937 cells co‐cultured with N‐EV and H‐EV measured by RT‐qPCR. (F) The expression of miR‐10b‐5p in U937 cells treated with miR‐10b‐5p‐mimic evaluated by RT‐qPCR. (G) The mRNA expression of iNOS, TNF‐α, Arg‐1, and IL‐10 in U937 cells treated with miR‐10b‐5p‐mimic assessed by RT‐qPCR. (H) Immunoblotting of protein expression of iNOS and Arg‐1 in U937 cells treated with miR‐10b‐5p‐mimic. (I) The expression of TNF‐α and IL‐10 in U937 cell supernatant treated with miR‐10b‐5p‐mimic detected by ELISA. (J) The proportion of CD11b^+^CD163^+^ cells in U937 cells treated with miR‐10b‐5p‐mimic evaluated by flow cytometry. (K) The proliferation in glioma U87 and A172 cells co‐cultured with miR‐10b‐5p‐ mimic or mimic‐NC transfected U937 cells tested by EDU. (L) Cell migration in U87 and A172 cells co‐cultured with miR‐10b‐5p‐mimic or mimic‐NC transfected U937 cells examined by Transwell. (M) Cell invasion in U87 and A172 cells co‐cultured with miR‐10b‐5p‐mimic or mimic‐NC transfected U937 cells examined by Transwell. *p < 0.05 versus brain tissues from non‐glioma or U937 cells treated with PBS or mimic‐NC. ^#^ *p* < 0.05 versus U937 treated with normal oxygen‐induced glioma‐derived EVs. ^&^ *p* < 0.05 versus U87 or A172 co‐cultured with U937 cells without any treatment. The experiment was repeated three times

Differential analysis of glioma miRNA expression dataset GSE65626 was performed to obtain 133 significantly differentially expressed miRNAs (Figure 2A). Further intersection of those significantly upregulated miRNAs with those expressed in blood EVs from the EVmiRNA database was implemented to finally obtain 2 candidate miRNAs, miR‐21‐5p, and miR‐10b‐5p (Figure 2B). Analysis of differential expression of these two miRNAs in microarray revealed a greater fold difference for miR‐10b‐5p (Table S2). RT‐qPCR displayed that elevation of miR‐10b‐5p occurred in glioma tissues (Figure 2C). Besides, higher miR‐10b‐5p level was observed in H‐EV than in N‐EV (Figure 2D). After co‐cultivating N‐EV and H‐EV with macrophages U937, we found that N‐EV or H‐EV could significantly increase miR‐10b‐5p in U937 cells, and miR‐10b‐5p level was upregulated in H‐EV‐treated U937 cells compared with N‐EV (Figure 2E).

Subsequently, we elevated miR‐10b‐5p level in U937 cells (Figure 2F). RT‐qPCR, Immunoblotting, and ELISA displayed that miR‐10b‐5p elevation decreased iNOS and TNF‐α expression but increased Arg‐1 and IL‐10 expression (Figure 2G–I). Flow cytometry revealed that miR‐10b‐5p‐mimic treatment boosted the proportion of CD11b^+^CD163^+^ cells (Figure 2J). The above results proved that miR‐10b‐5p could stimulate macrophages M2 polarization. Next, transfected U937 cells were co‐cultured with glioma U87 and A172 cells. It was evident that U87 and A172 cell proliferation, migration, and invasion were accelerated after co‐culture of U87 and A172 cells with miR‐10b‐5p‐mimic‐treated U937 cell (Figure 2K–M; Figure S1A–C).

We also suppressed the expression of miR‐10b‐5p in H‐EV for further validating the above assays (Figure S2). RT‐qPCR demonstrated that miR‐10b‐5p level was decreased after miR‐10b‐5p inhibition in H‐EVs (Figure S2A,B). Besides, miR‐10b‐5p inhibition in H‐EVs exhibited increase in iNOS and TNF‐α expression and decrease in Arg‐1 and IL‐10 levels (Figure S2C). Flow cytometry revealed that miR‐10b‐5p inhibition markedly reduced the proportion of CD11b^+^CD163^+^ cells in H‐EVs (Figure S2D). Moreover, miR‐10b‐5p inhibition reduced U937 cell proliferation, migration, and invasion of U87 and A172 cells (Figure S2E–G). The above results confirmed that hypoxic glioma cell‐derived EVs containing miR‐10b‐5p contributed to M2 macrophage polarization, thus promoting the progression of glioma.

### 3.3 MiR‐10b‐5p targets NEDD4L in macrophages

**FIGURE 3:**
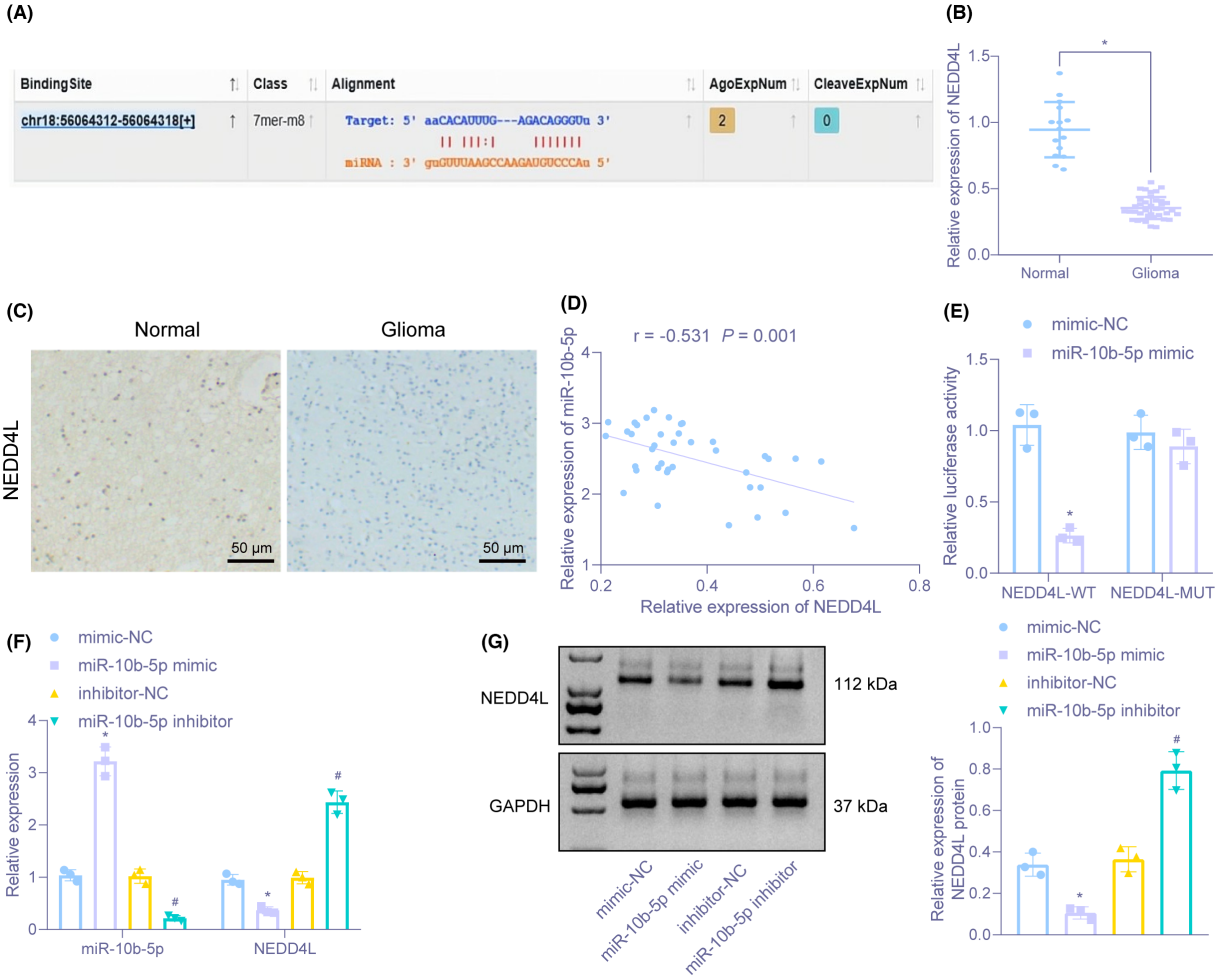
NEDD4L is negatively targeted by miR‐10b‐5p in macrophages. (A) The target site between miR‐10b‐5p and NEDD4L predicted through the online Starbase website. (B) The expression of NEDD4L in brain tissues of 40 cases of glioma and 15 cases of non‐glioma measured by RT‐qPCR. (C) The expression of NEDD4L in brain tissues of 40 cases of glioma and 15 cases of non‐glioma determined by immunohistochemistry (×200). (D) NEDD4L expression negatively correlated with miR‐10b‐5p expression revealed by Pearson correlation analysis. (E) Targeting relation between miR‐10b‐5p and NEDD4L identified by dual‐luciferase reporter gene assay. (F) The expression of miR‐10b‐5p and mRNA expression of NEDD4L in U937 cells after miR‐10b‐5p alteration assessed by RT‐qPCR. (G) Immunoblotting for determination of protein expression of miR‐10b‐5p and NEDD4L in U937 cells. *p < 0.05 versus brain tissues from patients with non‐glioma or NEDD4L 3′UTR‐WT co‐transfection with mimic‐NC. ^#^ *p* < 0.05 versus macrophages treated with inhibitor‐NC. The experiment was repeated three times

The target site of miR‐10b‐5p to the 3′UTR region of NEDD4L was predicted through the online Starbase website (Figure 3A). Studies have found that NEDD4L is an E3‐specific ubiquitin ligase belonging to the NEDD4 family, and that NEDD4L can ubiquitinate and degrade PIK3CA.^16^, ^18^ We identified reduced NEDD4L expression in brain tissues from glioma (Figure 3B,C). Pearson correlation analysis revealed inverse correlation between miR‐10b‐5p and NEDD4L in glioma samples (Figure 3D). Dual‐luciferase reporter gene assay presented that miR‐10b‐5p elevation declined luciferase signal of NEDD4L 3′UTR‐WT (Figure 3E). Besides, miR‐10b‐5p mimic treatment downregulated NEDD4L level while miR‐10b‐5p inhibitor treatment brought about opposite trend in U937 cells (Figure 3F,G). The above results illustrated that miR‐10b‐5p targeted NEDD4L and inversely regulated its expression in macrophages.

### 3.4 MiR‐10b‐5p promotes M2 polarization of macrophages to enhance oncogenic phenotypes of glioma cells by downregulating NEDD4L expression

**FIGURE 4:**
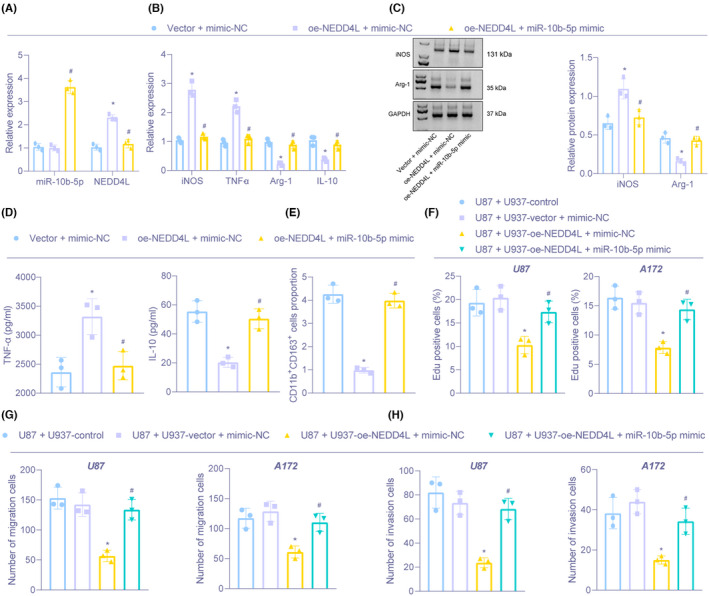
miR‐10b‐5p stimulates M2 polarization of macrophages to increase proliferation, migration, and invasion of glioma cells via downregulation of NEDD4L. (A) The expression of miR‐10b‐5p and NEDD4L in U937 cells after overexpression of miR‐10b‐5p and NEDD4L measured by RT‐qPCR. (B) The mRNA expression of iNOS, TNF‐α, Arg‐1, and IL‐10 in U937 cells after overexpression of miR‐10b‐5p and NEDD4L determined by RT‐qPCR. (C) The protein expression of iNOS and Arg‐1 in U937 cells after overexpression of miR‐10b‐5p and NEDD4L examined by Immunoblotting. (D) The expression of TNF‐α and IL‐10 assessed in supernatant of U937 cells after overexpression of miR‐10b‐5p and NEDD4L examined by ELISA. (E) The proportion of CD11b^+^CD163^+^ cells in U937 cells after overexpression of miR‐10b‐5p and NEDD4L evaluated by flow cytometry. (F) The proliferation in glioma U87 and A172 cells co‐cultured with transfected U937 cells assessed by EdU. (G) Cell migration in U87, and A172 cells co‐cultured with U937 cells tested by Transwell. H, Cell invasion in U87 and A172 cells co‐cultured with U937 cells tested by Transwell. *p < 0.05 versus U937 cells transfected with Vector and mimic‐NC or U87 or A172 co‐cultured with U937 cells treated without anything. ^#^ *p* < 0.05 versus U937 cells transfected with NEDD4L and mimic‐NC or U87 or A172 co‐cultured with U937 cells transfected with NEDD4L and mimic‐NC. The experiment was repeated three times

We then moved to study the effect of miR‐10b‐5p/NEDD4L on macrophage polarization. oe‐NEDD4L treatment elevated NEDD4L expression but exerted no change in miR‐10b‐5p expression, while further miR‐10b‐5p mimic treatment enhanced miR‐10b‐5p level and diminished NEDD4L expression in U937 cells (Figure 4A). RT‐qPCR, immunoblotting, and ELISA suggested that overexpression of NEDD4L reduced Arg‐1 and IL‐10 levels, but elevated iNOS and TNF‐α expression, while further miR‐10b‐5p mimic treatment brought about contrary findings (Figure 4B–D). Flow cytometry revealed that the proportion of CD11b^+^CD163^+^ cells was inhibited by upregulation of NEDD4L, but this trend was revoked via miR‐10b‐5p mimic (Figure 4E). As reflected by EDU and Transwell assays, U87 and A172 cell proliferation, migration, and invasion were suppressed through NEDD4L‐overexpressed U937 cells, which was neutralized by miR‐10b‐5p‐mimic (Figure 4F–H).

### 3.5 NEDD4L promotes ubiquitination degradation of PIK3CA in macrophages

**FIGURE 5:**
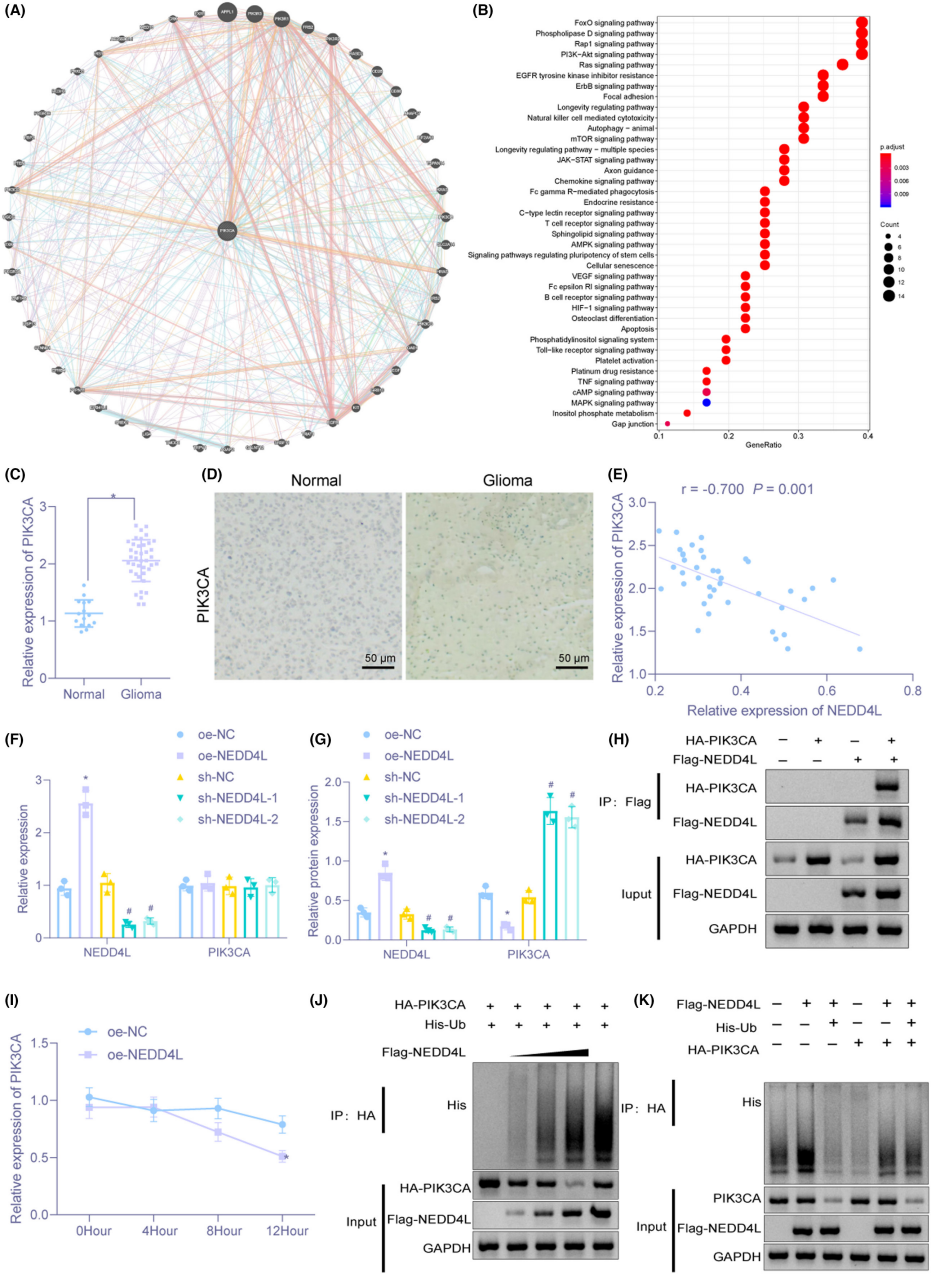
NEDD4L enhances ubiquitination and degradation of PIK3CA in macrophages. (A) PIK3CA‐related gene prediction. Circle center denotes PIK3CA gene, and others are predicted related genes. (B) KEGG pathway enrichment analysis of PIK3CA‐related genes. The *x*‐axis represents GeneRatio, the *y*‐axis represents the KEGG entry name, and the histogram on the right is color scale. (C) The expression of PIK3CA in brain tissues of 40 cases of glioma and 15 cases of non‐glioma examined by RT‐qPCR. (D) The expression of PIK3CA in brain tissues of 40 cases of glioma and 15 cases of non‐glioma assessed by immunohistochemistry (×200). (E) NEDD4L expression negatively correlated with PIK3CA expression identified by Pearson correlation analysis. (F) The mRNA expression of NEDD4L and PIK3CA in U937 cells evaluated by RT‐qPCR. (G) Immunoblotting for measurement of protein expression of NEDD4L and PIK3CA in U937 cells. (H) Interaction between NEDD4L and PIK3CA verified by Co‐IP assay. (I) The protein expression of NEDD4L and PIK3CA in U937 cells with 20–40 μg/ml CHX treatment determined by Immunoblotting. (J) The effect of NEDD4L on PIK3CA ubiquitination in HEK293T cells detected by IP assay. (K) The impact of NEDD4L on endogenous PIK3CA ubiquitination in HEK293T cells examined by IP assay. *p < 0.05 versus brain tissues from patients with non‐glioma or U937 cells transfected with oe‐NC. ^#^ *p* < 0.05 versus U937 cells transfected with sh‐NC. The experiment was repeated three times

Further prediction of PIK3CA‐related genes (Figure 5A) and KEGG pathway enrichment analysis of related genes (Figure 5B) manifested that PIK3CA‐related genes were mainly enriched in signaling pathways including PI3K/AKT. PIK3CA can be degraded by NEDD4L through ubiquitination to regulate the PI3K/AKT pathway.^24^ Therefore, we speculated that NEDD4L could regulate glioma development via PIK3CA and PI3K/AKT pathway. RT‐qPCR and immunohistochemistry displayed that PIK3CA level was higher in brain tissues from patients with glioma than in non‐glioma samples (Figure 5C,D). Pearson correlation analysis identified negative correlation between PIK3CA and NEDD4L expression (Figure 5E).

We overexpressed or silenced NEDD4L in U937 cells to investigate the regulation of NEDD4L on PIK3CA in macrophages. RT‐qPCR and Immunoblotting presented that PIK3CA protein expression was decreased after NEDD4L was overexpressed. NEDD4L silencing elevated the protein expression of PIK3CA. However, PIK3CA mRNA expression did not change significantly in macrophages after overexpressing or silencing NEDD4L (Figure 5F,G), indicating that NEDD4L could regulate PIK3CA at the protein level instead of mRNA level. To test whether NEDD4L could interact with PIK3CA, HA‐PIK3CA and/or Flag‐NEDD4L were overexpressed in HEK293T cells, and NEDD4L and PIK3CA were immunoprecipitated with Flag antibody, which depicted that PIK3CA and NEDD4L was interacted with each other (Figure 5H).

To further investigate ubiquitination regulation of PIK3CA by NEDD4L, 100 μM cycloheximide (CHX) was used to treat HEK293T cells to inhibit protein synthesis. Immunoblotting displayed that with the same time of treatment, the degradation rate of PIK3CA was elevated by overexpressing NEDD4L (Figure 5I), which showed that overexpression of NEDD4L could induce the degradation of PIK3CA. To detect whether NEDD4L promoted PIK3CA protein degradation by promoting ubiquitination of PIK3CA, we simultaneously overexpressed His‐Ub and HA‐PIK3CA. The results demonstrated that with the increasing amount of labeling of NEDD4L, PIK3CA ubiquitination was augmented (Figure 5J). NEDD4L was exogenously expressed in HEK293T cells, and exogenous expression of NEDD4L induced endogenous PIK3CA ubiquitination, but MG132 treatment could inhibit NEDD4L‐dependent PIK3CA ubiquitination and degradation (Figure 5K). Taken together, overexpression of NEDD4L could decrease the stability of PIK3CA in macrophages.

### 3.6 NEDD4L inactivates the PI3K/AKT pathway to inhibit M2 polarization of macrophages through PIK3CA

**FIGURE 6:**
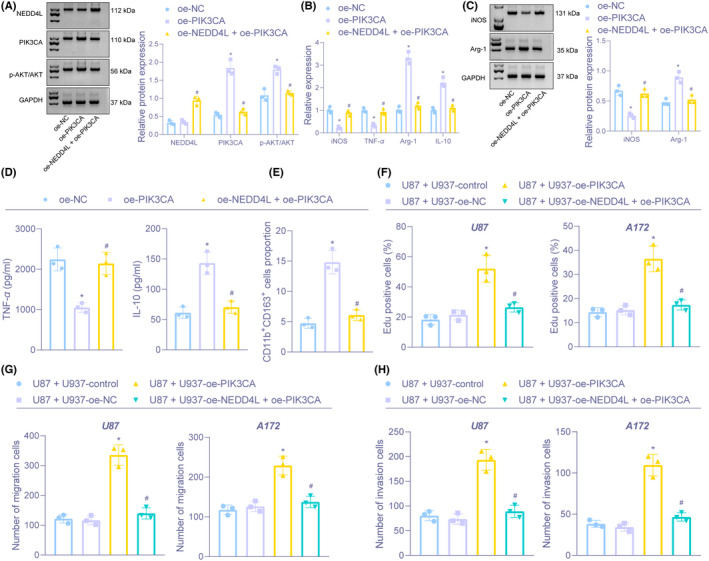
NEDD4L downregulates PIK3CA to inhibit the PI3K/AKT pathway, thus restraining M2 polarization of macrophages and glioma cell proliferation, migration, and invasion. (A) The protein expression of NEDD4L, PIK3CA, and AKT and phosphorylated AKT in U937 cells after overexpression NEDD4L and PIK3CA examined by Immunoblotting. (B) The mRNA expression of iNOS, TNF‐α, Arg‐1, and IL‐10 in U937 cells after overexpression NEDD4L and PIK3CA measured by RT‐qPCR. (C) Immunoblotting of protein expression of iNOS and Arg‐1 in U937 cells after overexpression NEDD4L and PIK3CA. (D) The expression of TNF‐α and IL‐10 in supernatant of U937 cells after overexpression NEDD4L and PIK3CA assessed by ELISA. (E) The proportion of CD11b^+^CD163^+^ cells in U937 cells after overexpression NEDD4L and PIK3CA evaluated by flow cytometry. (F) The proliferation in U87 and A172 cells co‐cultivated with U937 cells tested by EdU. (G) Cell migration in U87 and A172 cells co‐cultivated with U937 cells examined by Transwell. (H) Cell invasion in U87 and A172 cells co‐cultivated with U937 cells examined by Transwell. *p < 0.05 versus U937 cells transfected with oe‐NC or U87 or A172 co‐cultivated with U937 cells treated without anything. ^#^ *p* < 0.05 versus U937 cells transfected with oe‐PIK3CA or U87 or A172 co‐cultivated with U937 cells transfected with oe‐PIK3CA. The experiment was repeated three times

To examine the effect of NEDD4L on PI3K/AKT pathway, we performed immunoblotting and found that protein expression of PIK3CA and phosphorylated AKT were significantly increased; NEDD4L and AKT levels did not change after overexpressing PIK3CA. Additional oe‐NEDD4L treatment resulted in an increase in NEDD4L expression, decrease in PIK3CA and phosphorylated AKT expression, with no change in AKT expression in the presence of oe‐PIK3CA (Figure 6A).

We also found increase in Arg‐1 and IL‐10 levels and decline in iNOS and TNF‐α expression through overexpression of PIK3CA, which was abrogated by upregulating NEDD4L (Figure 6B–D). Flow cytometry documented that the proportion of CD11b^+^CD163^+^ cells was boosted by upregulated PIK3C, but the promotive role of PIK3C was inhibited by overexpressing of NEDD4L (Figure 6E).

Based on the results of EDU and Transwell assays, co‐culture of U87 and A172 cells with PIK3CA‐overexpressed U937 cells led to promoted proliferation, migration, and invasion in U87 and A172 cells, but further treatment with oe‐NEDD4L neutralized those effects (Figure 6F–H). The above results demonstrated that NEDD4L overexpression repressed the PI3K/AKT pathway through inhibition of PIK3CA to reduce M2 polarization of macrophages, thus restraining glioma cell growth.

### 3.7 H‐EVs overexpressing miR‐10b‐5p promotes macrophage M2 polarization and enhances the tumorigenic capacity of glioma cells in nude mice

**FIGURE 7:**
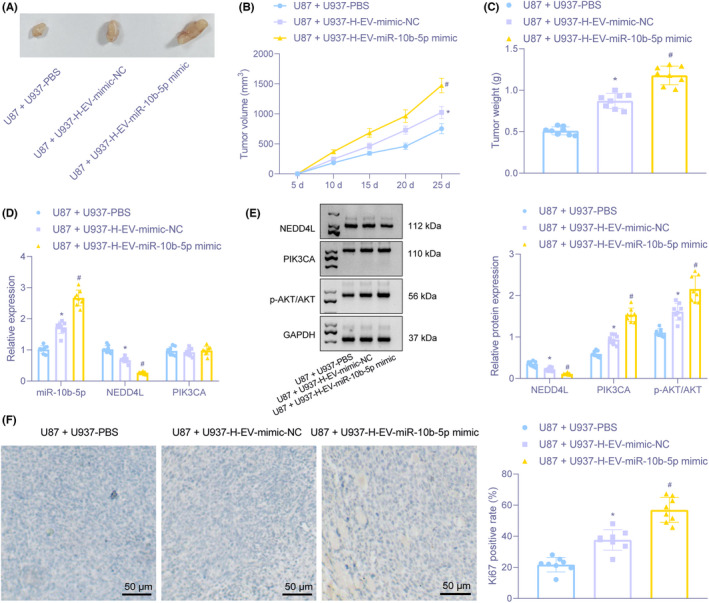
Macrophage accelerates the formation of glioma in nude mice through hypoxic glioma‐derived EVs carrying miR‐10b‐5p. (A) Images of subcutaneous tumor formation after U937 treated with hypoxic glioma cell‐derived EVs, and U87 cells were transplanted into nude mice (*n* = 8). (B) Curves of subcutaneous tumor growth in nude mice transplanted with U937 cells treated with hypoxic glioma cell‐derived EVs and U87 cells (*n* = 8). (C) Tumor weight of tumor in nude mice transplanted with U937 cells treated with hypoxic glioma cell‐derived EVs and U87 cells presented by histogram (*n* = 8). (D) RT‐qPCR for determination of mRNA expression of miR‐10b‐5p, NEDD4L, PIK3CA (*n* = 8). (E) The protein expression of NEDD4L, PIK3CA, and AKT and phosphorylated AKT in tumor of nude mice transplanted with U937 cells treated with hypoxic glioma cell‐derived EVs and U87 cells determined by Immunoblotting (*n* = 8). (F) The expression of Ki67 in tumor of nude mice transplanted with U937 cells treated with hypoxic glioma cell‐derived EVs and U87 cells examined by Immunohistochemistry (*n* = 8) (×200). *p < 0.05 versus tumors of nude mice transplanted with U87 cells and U937 treated with PBS. ^#^ *p* < 0.05 versus tumors of nude mice transplanted with U87 cells and U937 treated with H‐EV‐mimic‐NC

The above in vitro findings were also validated in vivo in the following. As shown in Figure 7A–C, mice injected with U87 + U937‐H‐EV‐mimic‐NC or U87 + U937‐H‐EV‐miR‐10b‐5p mimic had increased tumor volume and weight, especially the later. It was demonstrated that U937 cells treated with hypoxic glioma cell‐derived EVs harboring miR‐10b‐5p facilitated the progression of glioma.

As depicted in Figure 7D,E, mice transplanted with U87 cells and U937 treated with H‐EV‐miR‐10b‐5p mimic showed elevated miR‐10b‐5p, PIK3CA protein expression, p‐PI3K, and p‐AKT expression, but decreased NEDD4L, with unchanged PIK3CA mRNA expression and AKT protein expression. Immunohistochemistry displayed that Ki67 expression was increased in tumors from mice transplanted with U87 cells and U937 treated with H‐EV‐mimic‐NC in comparison with PBS treatment. Moreover, the expression of Ki67 in tumors from mice transplanted with U87 cells and U937 treated with H‐EV‐miR‐10b‐5p mimic was elevated versus those transplanted with U87 cells and U937 treated with H‐EV‐mimic‐NC (Figure 7F). The above results demonstrated that hypoxic glioma cell‐derived EVs carrying overexpressed miR‐10b‐5p accelerated tumor progression in nude mice.

## 4 DISCUSSION

**FIGURE 8:**
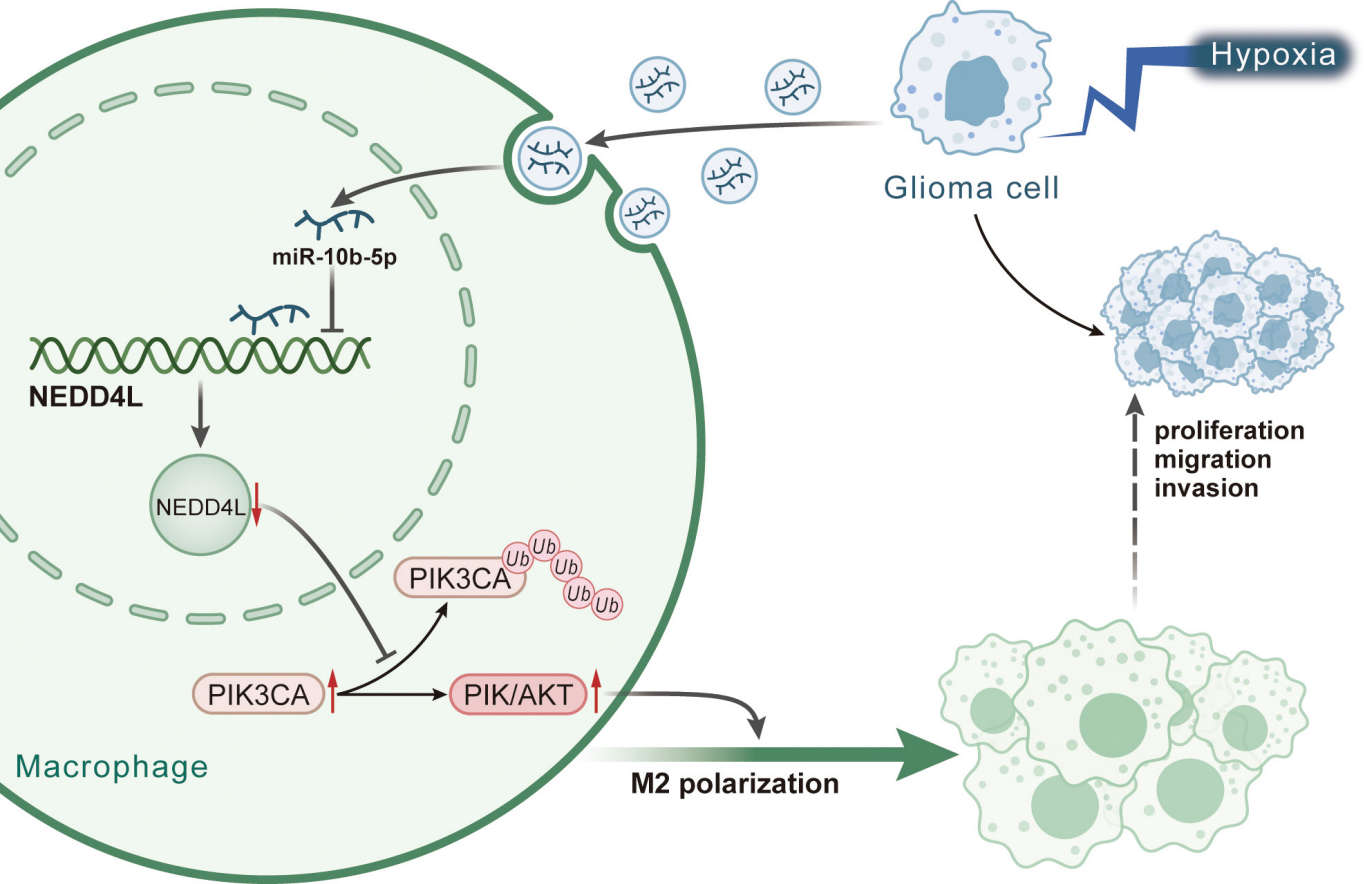
Regulatory mechanism of hypoxic glioma‐derived EVs in glioma. Hypoxic glioma‐derived EVs harboring miR‐10b‐5p into macrophages reduced NEDD4L expression, thus diminishing the promotion of NEDD4L on the ubiquitylation and degradation of PIK3CA and increasing PIK3CA expression to activate the PI3K/AKT pathway. In this way, M2 polarization of macrophages was promoted, ultimately potentiating the proliferation, migration, and invasion of glioma cells

Glioma‐derived EVs can carry biomolecules such as miRNAs and function as potential tumor‐related biomarkers for developing new anti‐glioma therapy.^27^ In the present study, we mainly focused on the regulatory significance of hypoxic glioma‐derived EVs carrying miR‐10b‐5p in M2 macrophage polarization and the progression of glioma. Our study indicated the tumor‐promoting capacity of hypoxic glioma cell‐derived EVs harboring miR‐10b‐5p for glioma via upregulated PIK3CA and activated PI3K/AKT pathway through downregulating NEDD4L (Figure 8).

Our initial finding was that hypoxic glioma‐derived EVs could promote macrophages M2 polarization, as evidence by decreased expression of iNOS and TNF‐α as well as elevated expression of Arg‐1 and IL‐10. Macrophages are known for their classic M1 polarization or alternative M2 polarization.^28^ M1 macrophages secrete proinflammatory factors such as TNF‐α and iNOS, while M2 macrophages secrete anti‐inflammatory factors including Arg‐1 and IL‐10.^29^ Concurred with our findings, glioblastoma‐derived EVs can modify the phenotype of macrophages and result in activated M2 macrophages.^30^ Besides, our study demonstrated that miR‐10b‐5p was upregulated in glioma tissues, hypoxic glioma‐derived EVs and macrophages co‐cultured with EVs. Interestingly, miR‐10b‐5p elevation occurs overexpressed in high‐grade glioma tissues.^31^ In a recent research, downregulation of miR‐10b‐5p could augment anti‐tumor effect of the Kv channel blocker 4‐aminopyridine by promoting glioma cell apoptosis.^32^ Glioma‐derived EVs containing miRNA enable to trigger angiogenesis.^33^ In addition, hypoxic glioma‐derived EVs delivering microRNA‐1246 could stimulate M2 macrophage polarization.^34^ Intriguingly, hypoxic glioma cell‐derived EVs could deliver miR‐10b‐5p to normoxic glioma cells to enhance aggressive tumor biology of cells.^20^ Similar to this prior research, we also found that hypoxic glioma‐derived EVs carrying miR‐10b‐5p promoted M2 macrophage polarization and enhanced malignant phenotype of glioma cells.

Moreover, we found that NEDD4L was targeted by miR‐10b‐5p and that NEDD4L exhibited a low expression in glioma tissues. Wang et al. have reported that miR‐10b‐5p targets IQGAP2 and downregulates its expression to elevate triple‐negative breast cancer cell oncogenic phenotypes.^35^ Additionally, it has been illustrated that miR‐513a‐5p desensitizes glioma cells to temozolomide by targeting NEDD4L.^36^ These findings were partially consistent with our study that miR‐10b‐5p downregulated NEDD4L expression to promote M2 macrophages polarization so as to accelerate malignant phenotypes of glioma cells. NEDD4L serves as a tumor‐suppressor gene in a variety of cancer. For instance, downregulation of NEDD4L occurs in non‐small cell lung cancer and its elevation hampered aggressive tumor biology of cells.^37^ Also, Dong‐Eun Lee et al. have revealed the evidence that NEDD4L exerts inhibitory function on the growth of pancreatic cancer cells via suppression of autophagy and mitochondrial metabolism.^38^ More importantly, NEDD4L is downregulated in glioma cells, which is linked to aggressiveness and poor prognosis of malignant glioma.^23^ The upregulation of NEDD4L had tumor‐suppressing properties in glioma.^36^ Moreover, results from the current study showed that the ubiquitination degradation of PIK3CA was promoted by NEDD4L in macrophages. PIK3CA is a main regulator of PI3K/AKT pathway. Mutation of PIK3CA has been confirmed in various cancers, which is considered to serve as an oncogene.^39^ Mutations of PIK3CA lead to neuronal hyperactivity during formation of glioma.^40^ Our study added to the growing evidence for the activation of PIK3CA in glioma tissues. Activation of PI3K/AKT pathway is capable of enhancing glioma progression and promoting chemotherapy resistance.^41^ Cell invasion in glioma is attributed by activated PI3K/AKT pathway.^42^ Meanwhile, our results further confirmed that NEDD4L inhibits the PI3K/AKT pathway through PIK3CA to inhibit M2 polarization of macrophages, thereby restraining cell malignant phenotypes in glioma.

Taken together, our study supported that miR‐10b‐5p could be encapsulated by glioma cell‐derived EVs into macrophages and provided evidence suggesting that hypoxic glioma cell‐derived EVs carrying miR‐10b‐5p could stimulate M2 polarization of macrophages and accelerate glioma cell proliferation, migration, and invasion through activated PI3K/AKT pathway by increasing expression of PIK3CA via downregulation of NEDD4L expression. Thus, the current study yields a more profound understanding of glioma, underscoring a potential target against glioma in the future.

## CONFLICT OF INTEREST

The authors declare no conflicts of interest.

## Supporting information


Figure S1

Figure S2

Table S1
Table S2

## Data Availability

The data that support the findings of this study are available in this article and its supplementary material.
