# Supplementary figures and images for: Hypoxic glioma‐derived extracellular vesicles harboring MicroRNA‐10b‐5p enhance M2 polarization of macrophages to promote the development of glioma
Source: CNS Neurosci Ther. 2022 Sep 2;28(11):1733–47. doi: 10.1111/cns.13905 (PMC9532931; doi:10.1111/cns.13905)

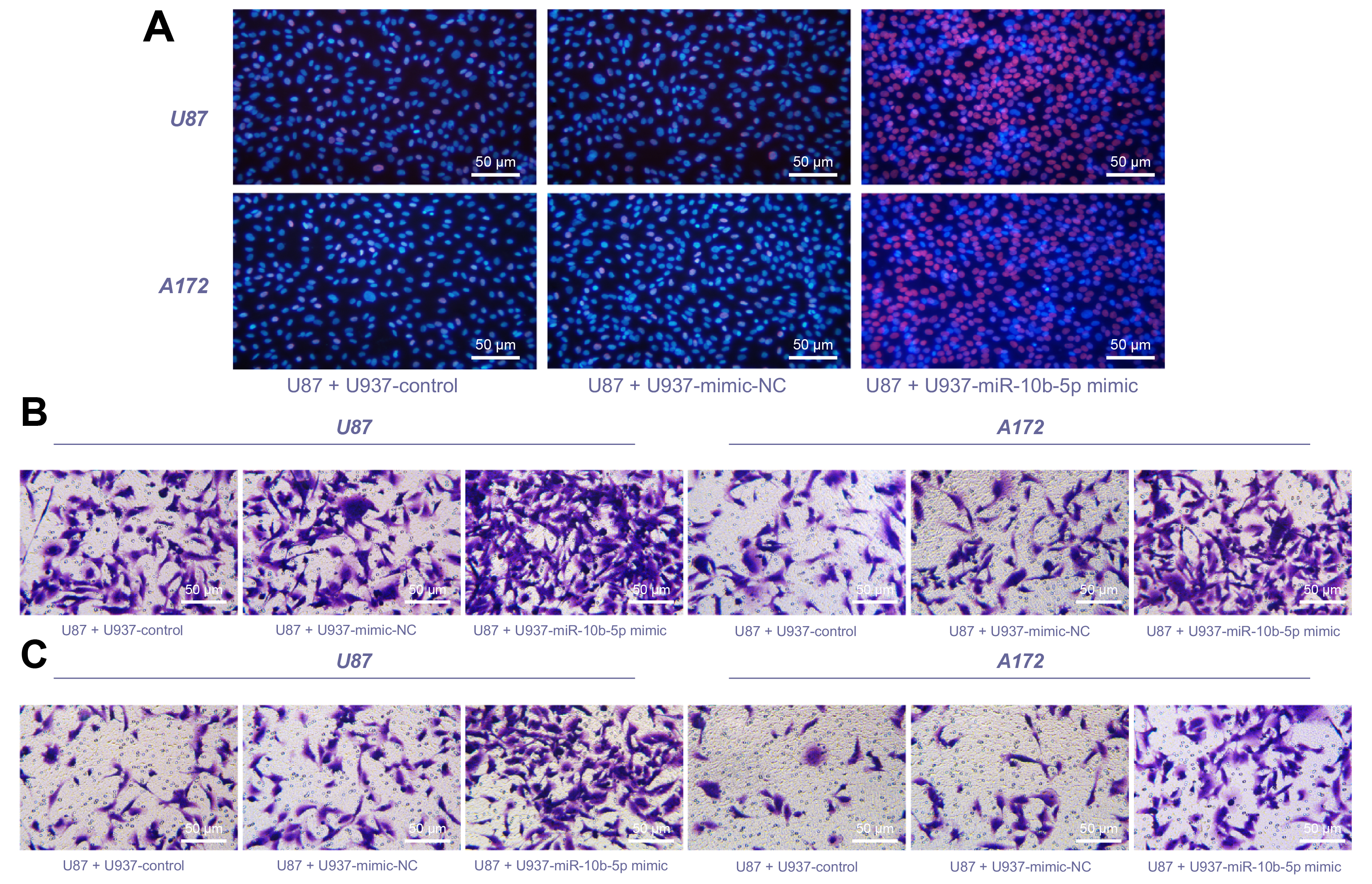

Supplement: Supplementary file 1 — Figure S1 [file CNS-28-1733-s002.jpg]

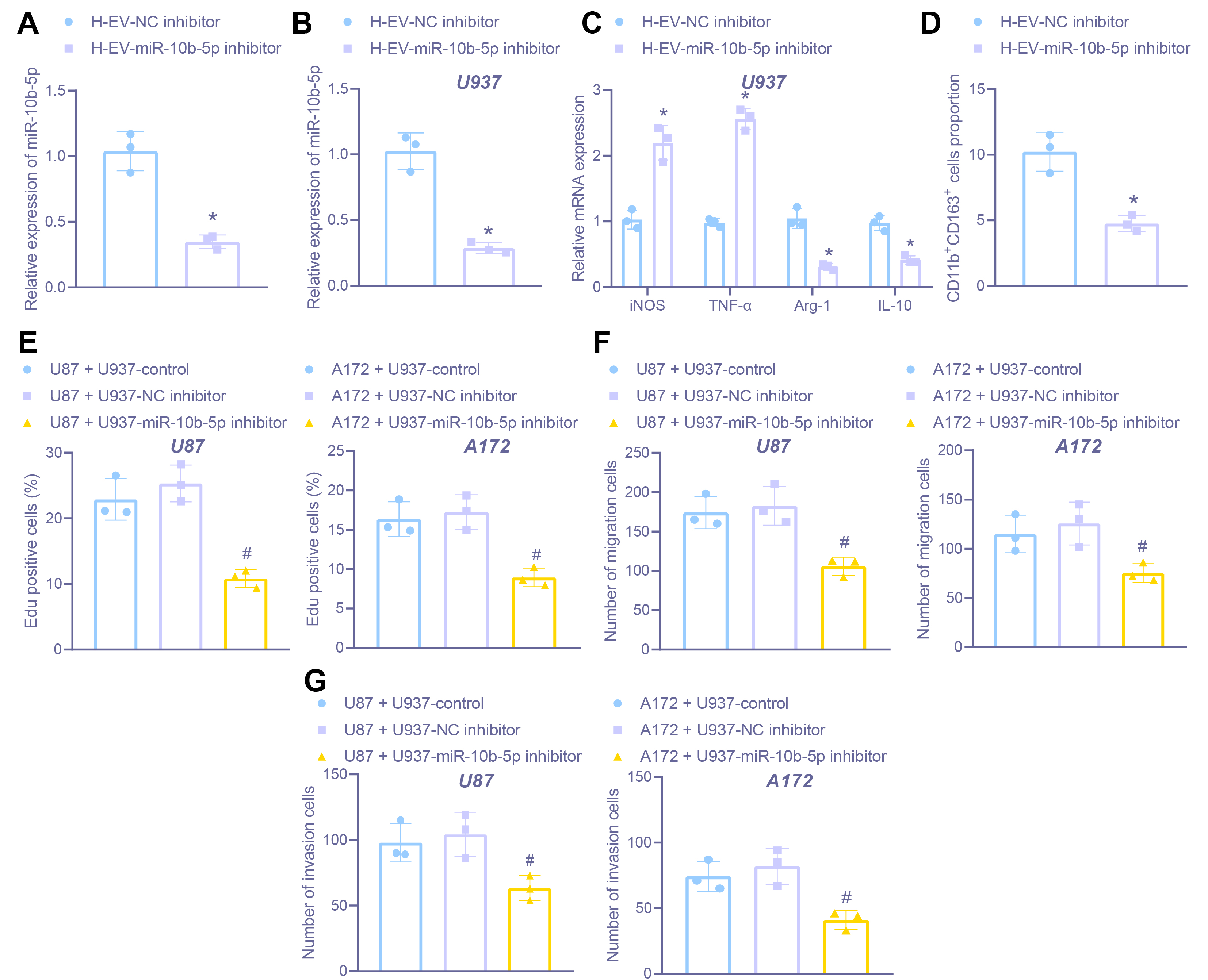

Supplement: Supplementary file 2 — Figure S2 [file CNS-28-1733-s001.jpg]
