# Supplementary material for: Hypoxic glioma‐derived extracellular vesicles harboring MicroRNA‐10b‐5p enhance M2 polarization of macrophages to promote the development of glioma
Source: CNS Neurosci Ther. 2022 Sep 2;28(11):1733–47. doi: 10.1111/cns.13905 (PMC9532931; doi:10.1111/cns.13905)
Supplement: Supplementary file 3 — Table S1 Table S2 [file CNS-28-1733-s003.docx]

**Supplementary Table 1** Primer sequences for RT-qPCR

| Gene | Primer sequence (5’-3’) |
| --- | --- |
| NEDD4L | F: TCCAATGGTCCTCAGCTGTTTA |
|  | R: ATTTTCCACGGCCATGAGA |
| miR-10b-5p | F: GTGTTTAAGCCAAGATGTCCCAT |
|  | R: TACCCTGTAGAACCGAATTTGTG |
| iNOS | F: universal primer |
|  | R: TTCAGTATCACAACCTCAGCAAG |
| Arg-1 | F: GTGGAAACTTGCATGGACAAC |
|  | R: AATCCTGGCACATCGGGAATC |
| TNF-α | F: GAGGCCAAGCCCTGGTATG |
|  | R: CGGGCCGATTGATCTCAGC |
| IL-10 | F: GACTTTAAGGGTTACCTGGGTTG |
|  | R: TCACATGCGCCTTGATGTCTG |
| PIK3CA | F: CCACGACCATCATCAGGTGAA |
|  | R: CCTCACGGAGGCATTCTAAAGT |
| U6 | F: CTCGCTTCGGCAGCACA |
|  | R: universal primer |
| GAPDH | F: ATCAAGAAGGTGGTGAAGCAGG |
|  | R: CGTCAAAGGTGGAGGAGTGG |

Note: RT-qPCR, reverse transcription quantitative polymerase chain reaction; NEDD4L, neuronal precursor cell expressed developmentally down-regulated 4-like; miR-30a-5p, microRNA-30a-5p; iNOS, inducible nitric oxide synthase; Arg-1, arginase 1; TNF-α, tumor-necrosis factor-α; IL-10, Interleukin 10; PIK3CA, Phosphatidylinositol-4, 5-Bisphosphate 3-Kinase; GAPDH, glyceraldehyde-3-phosphate dehydrogenase; F, forward; R, reverse.

**Supplementary Table 2** Analysis of the differential expression of these two miRNAs in microarray

| symbol | logFC | *p*.Value | adj.*p*.Val |
| --- | --- | --- | --- |
| hsa-miR-21-5p | 3.957104889 | 2.56E-05 | 0.018275956 |
| hsa-miR-10b-5p | 4.770815685 | 4.88E-05 | 0.019847509 |
